# Supplementary material for: Reconciling Mining with the Conservation of Cave Biodiversity: A Quantitative Baseline to Help Establish Conservation Priorities
Source: PLoS One. 2016 Dec 20;11(12):e0168348. doi: 10.1371/journal.pone.0168348 (PMC5173368; doi:10.1371/journal.pone.0168348)
Supplement: S1 Dataset — (ZIP) [file pone.0168348.s002.zip › Taxa/Serra Norte/SN_2013/N5S/Lista N5S-78.pdf]

CAVIDADE N5S-0078

Classe	Ordem	Fam/Outros	Gên/Outros	Espécie	S-U	U-S	Zon.
Arachnida	Acari	(Oribatida)		sp.4		X	P
Arachnida	Acari	Cunaxidae		sp.1	X		P
Arachnida	Acari	Penthaleidae		sp.21	X		P
Arachnida	Amblypygi	Phrynidae	*Heterophrynus*	*longicornis*		3	E
Arachnida	Araneae	Araneidae		jovem	X		E
Arachnida	Araneae	Corinnidae	*Tupirinna*	sp.1	2		E
Arachnida	Araneae	Ctenidae	*Phoneutria*	*reidyi*		9	E
Arachnida	Araneae	Drymusidae	*Drymusa*	*spelunca*	X	X	P
Arachnida	Araneae	Mysmenidae		jovem	X		E
Arachnida	Araneae	Ochyroceratidae		jovem		X	
Arachnida	Araneae	Pholcidae	*Mesabolivar*	*aurantiacus*		X	E/P
Arachnida	Araneae	Pholcidae	*Mesabolivar*	sp.1	X		E
Arachnida	Araneae	Salticidae		jovem	X		E/P
Arachnida	Araneae	Scytodidae	*Scytodes*	sp.1		1	P
Arachnida	Araneae	Tetrablemmidae	*Matta*	sp.1		X	P
Arachnida	Araneae	Theridiidae		jovem	X		E
Arachnida	Araneae	Theridiosomatidae	*Plato*	sp.1		X	P
Arachnida	Araneae	Theridiosomatidae	*Plato*	sp.1	X	X	P
Arachnida	Araneae	Uloboridae		sp.2	X		E
Arachnida	Opiliones	Sclerosomatidae	*Prionostema*	sp.1		2	P
Arachnida	Pseudoscorpiones	Chthoniidae	*Pseudochthonius*	sp.1	X	X	P
Chilopoda	Scolopendromorpha	Scolopendridae	*Otostigmus*	sp.1	1		
Diplopoda	Polydesmida	Chelodesmidae		sp.3		1	P
Diplopoda	Polydesmida	Pyrgodesmidae		jovem	X		P
Entognatha	Collembola	Entomobryidae		sp.3	X		P
Entognatha	Collembola	Entomobryidae		sp.6	X		E
Entognatha	Collembola	Paronellidae		sp.1	X	X	P
Entognatha	Diplura	Campodeidae		sp.1	X		P
Entognatha	Diplura	Japygidae		sp.1		X	P
Insecta	Blattodea	Blattellidae		sp.1	2	1	E
Insecta	Coleoptera	Carabidae		sp.5		X	E
Insecta	Coleoptera	Cryptophagidae		sp.1	X		P
Insecta	Coleoptera	Endomychidae		sp.1		X	E
Insecta	Coleoptera	Staphylinidae	Pselaphinae	sp.1		X	P
Insecta	Coleoptera	Staphylinidae	Pselaphinae	sp.15	X		P
Insecta	Coleoptera	Staphylinidae		sp.7		X	P
Insecta	Diptera	Cecidomyiidae	Cecidomyiinae	sp.	X	X	E/P
Insecta	Diptera	Limoniidae		sp.	X		P
Insecta	Diptera	Psychodidae	Phlebotominae	sp.	X	X	E/P
Insecta	Diptera	Sciaridae		sp.	X		P
Insecta	Diptera	Tipulidae	Tipulinae	sp.	X	X	E/P
Insecta	Heteroptera	Dipsocoroidea		sp.	X		P
Insecta	Heteroptera	Nabidae		sp.		X	P
Insecta	Heteroptera	Reduviidae	Reduviinae	sp.		1	P
Insecta	Homoptera	Cixiidae		sp.3	X		E
Insecta	Hymenoptera	Formicidae	*Camponotus*	sp.2	X	X	E/P
Insecta	Hymenoptera	Formicidae	*Gnamptogenys*	sp.1	X		E
Insecta	Hymenoptera	Formicidae	Myrmicinae	sp.1	X		E
Insecta	Hymenoptera	Formicidae	*Pachycondyla*	sp.1	X		

Insecta	Hymenoptera	Formicidae	*Pachycondyla*	sp.2	X		P
Insecta	Hymenoptera	Formicidae	*Paratrechina*	sp.1	X	X	P
Insecta	Hymenoptera	Formicidae	*Paratrechina*	sp.2	X		E
Insecta	Hymenoptera	Formicidae	*Pheidole*	sp.3		X	E
Insecta	Hymenoptera	Vespidae		sp.3	X		P
Insecta	Isoptera	Termitidae	*Nasutitermes*	sp.	X	X	E/P
Insecta	Lepidoptera	Roeslerstammiidae		sp.	X	X	P
Insecta	Orthoptera	Phalangopsidae	*Paraclodes*	sp.	3	1	E/P
Insecta	Orthoptera	Phalangopsidae	*Phalangopsis*	sp.		9	E/P
Insecta	Psocoptera			jovem		X	P
Gastropoda	Pulmonata	Systrophiidae	*Happia*	sp.	X		P
Malacostraca	Decapoda	Pseudothelphusidae		sp.1		1	E
Malacostraca	Isopoda	Dubioniscidae		sp.1		X	P
Amphibia	Anura			sp.	4	1	E/P
Mammalia	Chiroptera	Emballonuridae	*Peropteryx*	*kappleri*	2	1	E/P
Mammalia	Chiroptera	Emballonuridae	*Peropteryx*	*macrotis*		1	E
